# Lassa Virus Seroprevalence in Sibirilia Commune, Bougouni District, Southern Mali

**DOI:** 10.3201/eid2204.151814

**Published:** 2016-04

**Authors:** Nafomon Sogoba, Kyle Rosenke, Jennifer Adjemian, Sory Ibrahim Diawara, Ousmane Maiga, Moussa Keita, Drissa Konaté, Abdoul Salam Keita, Ibrahim Sissoko, Matt Boisen, Diana Nelson, Darin Oottamasathien, Molly Millett, Robert F. Garry, Luis M. Branco, Sékou F. Traoré, Seydou Doumbia, Heinz Feldmann, David Safronetz

**Affiliations:** University of Sciences, Techniques and Technologies of Bamako, Bamako, Mali (N. Sogoba, S.I. Diawara, O. Maiga, M. Keita, D. Konaté, A.S. Keita, I. Sissoko, S.F. Traoré, S. Doumbia);; National Institutes of Health, Hamilton, Montana, USA (K. Rosenke, H. Feldmann, D. Safronetz);; National Institutes of Health, Bethesda, Maryland, USA (J. Adjemian);; Corgenix Medical Corporation, Inc., Broomfield, Colorado, USA (M. Boisen, D. Nelson, D. Oottamasathien, M. Millett);; Tulane School of Medicine, New Orleans, Louisiana, USA (R.F. Garry);; Zalgen Labs LLC, Germantown, Maryland, USA (L.M. Branco);; Public Health Agency of Canada, Winnipeg, Manitoba, Canada (D. Safronetz)

**Keywords:** Lassa fever, Lassa virus seroprevalence, arenavirus, viral hemorrhagic fever, West Africa, southern Mali, emerging infectious diseases, viruses, zoonoses

## Abstract

Lassa virus (LASV) is endemic to several nations in West Africa. In Mali, LASV was unknown until an exported case of Lassa fever was reported in 2009. Since that time, rodent surveys have found evidence of LASV-infected *Mastomys natalensis* rats in several communities in southern Mali, near the border with Côte d’Ivoire. Despite increased awareness, to date only a single case of Lassa fever has been confirmed in Mali. We conducted a survey to determine the prevalence of LASV exposure among persons in 3 villages in southern Mali where the presence of infected rodents has been documented. LASV IgG seroprevalence ranged from 14.5% to 44% per village. No sex bias was noted; however, seropositivity rates increased with participant age. These findings confirm human LASV exposure in Mali and suggest that LASV infection/Lassa fever is a potential public health concern in southern Mali.

## Abstract 2

The high rate documented in this study highlights the need for increased surveillance.

Lassa Virus Seroprevalence, Mali

Lassa virus (LASV) (family *Arenaviridae*, genus *Arenavirus*) is the etiologic agent of Lassa fever (LF), a viral hemorrhagic fever first documented in 1969 during an outbreak on the Jos Plateau in Nigeria (*1*). In humans, LASV infection is characterized by a variety of clinical manifestations that can range from apparently asymptomatic or mild disease to severe disease consisting of multiorgan failure (*2*,*3*). As much as 80% of infected persons are believed to experience mild disease, whereas 20% exhibit noteworthy and often severe clinical indicators that require medical attention (*4*). An estimated 300,000 LASV infections occur in West Africa each year, resulting in ≈5,000 deaths (*5*). Infection during pregnancy, especially during the third trimester, is particularly severe; estimated maternal mortality rates are 20%, and fetal mortality rates are ≈100% (*6*–*8*). As are most arenaviruses, LASV is maintained in nature in rodent hosts, specifically, the multimammate rat (*Mastomys natalensis*) (*9*). Most commonly, contact with infectious rodents or ingestion/inhalation of virus-laden particles is the source of human infection. Person-to-person transmission is also well documented and can result in outbreaks, especially in nosocomial settings, leading to mortality rates in >50% (*7*).

Historically, LASV has been considered endemic to 2 geographic areas of West Africa: 1) Sierra Leone, Guinea, and Liberia; and 2) Nigeria. However, in recent years, an increased region of LASV endemicity has been suggested, which includes adjoining countries and areas farther north than previously suggested (*10*,*11*). In 2000, a German citizen received a diagnosis of LF after traveling through Ghana, Côte d’Ivoire, and Burkina Faso (*12*). More recently, cases of LF have been identified in Ghana (*13*), and the presence of LASV-infected rodents has been documented in Côte d’Ivoire (*14*).

In a similar situation, LF was unknown in Mali until February 2009, when a young British man was medically evacuated to London after a 10-day history of fever (*15*). The infection was initially diagnosed as *Plasmodium falciparum* malaria, even though the patient did not respond to treatment for malaria. He died on arrival in London, and a postmortem diagnosis of LASV infection was confirmed by molecular techniques. In response to this case, rodent surveys were conducted in the village of Soromba (rural commune of Sibirila, Bougouni district, Mali), where the man was living and working when he became ill. The initial surveys found that 25% of *M. natalensis* rats had molecular evidence of active LASV infection, which was confirmed by virus isolation and sequence analysis (*16*). Similar studies conducted across Mali suggest that LASV is restricted to the southern tip of the country, in several villages near the border of Côte d’Ivoire (*17*). On average, 20% of peridomestic *Mastomys* rodents collected in these villages had serologic or molecular evidence of LASV infection, with peak prevalence rates >50%.

Given the infection rates observed in rodents living in close proximity to humans in many villages in southern Mali, it seems likely that humans are frequently exposed to LASV infection and that LF may develop. Nevertheless, despite increased recognition of LF in Mali, to date no outbreaks have occurred, and the 2009 exported case remains the only confirmed human LASV infection contracted in Mali. Reports of a second case of LF associated with the British citizen are unconfirmed. Verbal accounts indicate that shortly after he was evacuated, his housekeeper and cook also fell ill and died. Samples were not collected for testing, in part because malaria was suspected. To better understand the risk for human LASV infection in southern Mali, we conducted a serologic survey of inhabitants of 3 villages within the rural commune of Sibirila to determine the proportion of persons who had been exposed to LASV. 

## Materials and Methods

### Ethics Statement

**Ethical approval for research on human subjects was obtained from the independent institutional research boards of the Faculty of Medicine and Pharmacy of the University of Sciences, Techniques and Technologies of Bamako, Mali, and the US National Institutes of Health. Before we conducted these studies, permission was granted from regional health professionals as well as from village elders and chiefs.** Research on samples from human subjects was conducted in accordance with the policies and regulations of the National Institutes of Health and in adherence with the principles of the Belmont Report (1979) (http://www.hhs.gov/ohrp/humansubjects/guidance/belmont.html).

### Study Setting

**Figure 1:**
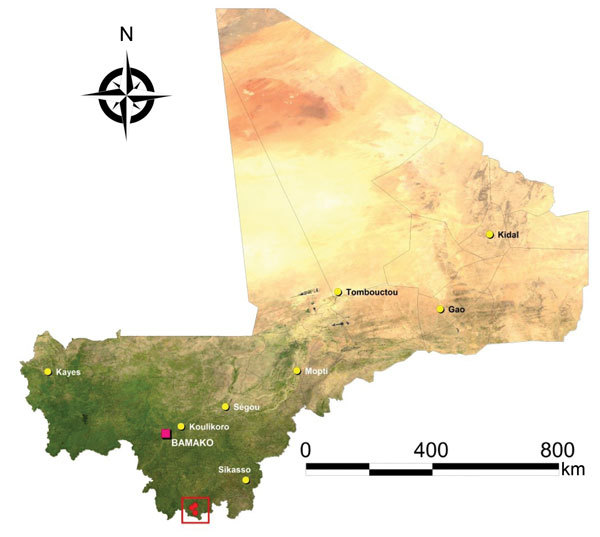
Study sites for assessment of Lassa virus seroprevalence in humans, southern Mali, 2015. The 3 villages of Soromba, Banzana, and Bamba (within red square) in Sibirilia commune, Bougouni district, were selected on the basis of previous identification of Lassa virus–infected rodents in peridomestic settings.

On the basis of findings from our previous rodent surveys in southern Mali, we selected the villages of Soromba **(10°35′21′′N, 07°09′21′′W), Bamba (10°22′59′′N, 07°09′06′′W), and Banzana (10°31′26′′N, 07°14′53′′W) (*17*) (**Figure 1**;** Technical Appendix Figure 1**). Soromba is the most likely exposure site of the only known case of LF in Mali and, along with Bamba, has the highest LASV prevalence documented in peridomestic rodents (***16**,**17***). Banzana is a nearby village with a low prevalence of LASV-infected rodents. According to a recent national census, the populations of Soromba, Bamba, and Banzana were 855, 1,751, and 4,822 persons, respectively. Samples were collected over 10 consecutive days in February 2015.**

### Consent and Enrollment

**Enrollment criteria for this study were the following: a participant must have been a healthy person >6 months of age with no febrile disease reported in the previous month, who (or whose parents) had resided in the community for at least 12 months. Oral and written consent** were **obtained from all participants in the study before their enrollment.** If the participant was illiterate, consent was granted in the presence of a literate witness of his or her choice or a trusted community member assigned by the community. **Consent for participants <18 years of age was given by parents or guardians. The study procedures and goals were explained to the participants in the local dialect (Bambara). In addition, all potential participants were given handouts explaining the study, and persons were encouraged to further discuss possible enrollment with trusted members of the community, primarily teachers and nurses. When possible, enrollees were given at least 24 hours to decide whether to voluntarily participate. The goal for this study was to enroll 600 participants, 200 per village.**

### Biometrics and Sample Collection

**Prior to sample collection, enrollees were given a physical examination by a licensed Malian physician. Participants with enlarged spleens, suspected of having an asymptomatic malaria infection, were tested with a rapid diagnostic test, and those whose test results were positive received treatment according to local guidelines. Also, children with suspected vitamin deficiencies were given supplements, and adults with varying medical conditions received treatment as required. Questionnaires were verbally administered to each participant >12 years of age; questions were asked about previous febrile diseases with or without hemorrhagic manifestations, rodent sightings/infestations in dwellings, and possible consumption of rodents. After the initial examinations, ≈1 mL of whole blood was collected by fingerstick into an EDTA-treated microtube by a certified Malian laboratory technician.**

### Sample Processing and Testing

**Within 1 h of blood collection, plasma was separated from whole blood by centrifugation, transferred to a cryovial, and frozen on liquid nitrogen. Samples were transported to a climate-controlled laboratory at the University of Bamako in dry shippers within 10 d of collection for serologic testing. An ELISA was used to screen samples for IgM and IgG reactive to a recombinant LASV nucleocapsid antigen derived from LASV Josiah (**ReLASV; Corgenix Medical Corporation, Inc., Broomfield, CO, USA) (*18*,*19*). **The kits are produced under a Corgenix Quality System (compliant with FDA regulations) (**http://www.corgenix.com/news-releases/corgenix-and-viral-hemorrhagic-fever-consortium-release-new-findings-from-sierra-leone-lassa-virus-program/#sthash.P7nnRrCI.dpuf**). The kits have been thoroughly evaluated for the detection of LASV antibodies in patients at the LF ward of the Kenema Government Hospital in Sierra Leone (L.M. Branco, M. Boisen, unpub. data) and have previously been used to detect anti-LASV antibodies in rodents collected in southern Mali (***17***). Optimized LASV ELISA methods developed at Kenema Government Hospital were used for testing the samples. In brief, serum specimens were initially screened at a 1:100 dilution in sample diluent. Samples were incubated on the ELISA plate at room temperature (≈25°C) for 30 min, after which they were washed 3 times with wash buffer on a mechanical plate washer. A peroxidase-labeled secondary antibody against human IgG or IgM was then added to each well, and samples were again incubated and washed as above. Peroxidase substrate (3, 3′, 5, 5′-tetramethylbenzidine, 100 μL/well) was added to each well and incubated at room temperature in the dark for ≈10 min after which a stop solution (0.16 M sulfuric acid, 100 μL/well) was added. Color development was assessed on a mechanical plate reader at 450 nm. A baseline for the assay was established by testing 374 serum samples collected from citizens of Mali who lived well outside the known region where LASV was endemic. Serum samples collected in the current study were considered serologically positive if they yielded optical densities (OD_450_) >2 SDs above the baseline value (>0.8). All reactive samples were titrated by 4-fold dilutions to determine the final titer. Serologic testing was done on blinded samples.**

### Statistical Analysis

Univariate generalized log-binomial regression models were used to estimate risk ratios (RRs) and 95% CIs and to identify factors that were significantly (p<0.05) associated with IgG seropositivity. Variables significant in univariate models were evaluated in multivariate models to assess for potential confounding effects. Univariate logistic regression models were used to identify significant factors associated with the expectedly rarer outcome of IgM seropositivity and estimate odds ratios and associated CIs. All regression analyses were conducted by using SAS version 9.3 (SAS Institute, Inc., Cary, NC, USA).

## Results 

### Study Demographics

**Figure 2:**
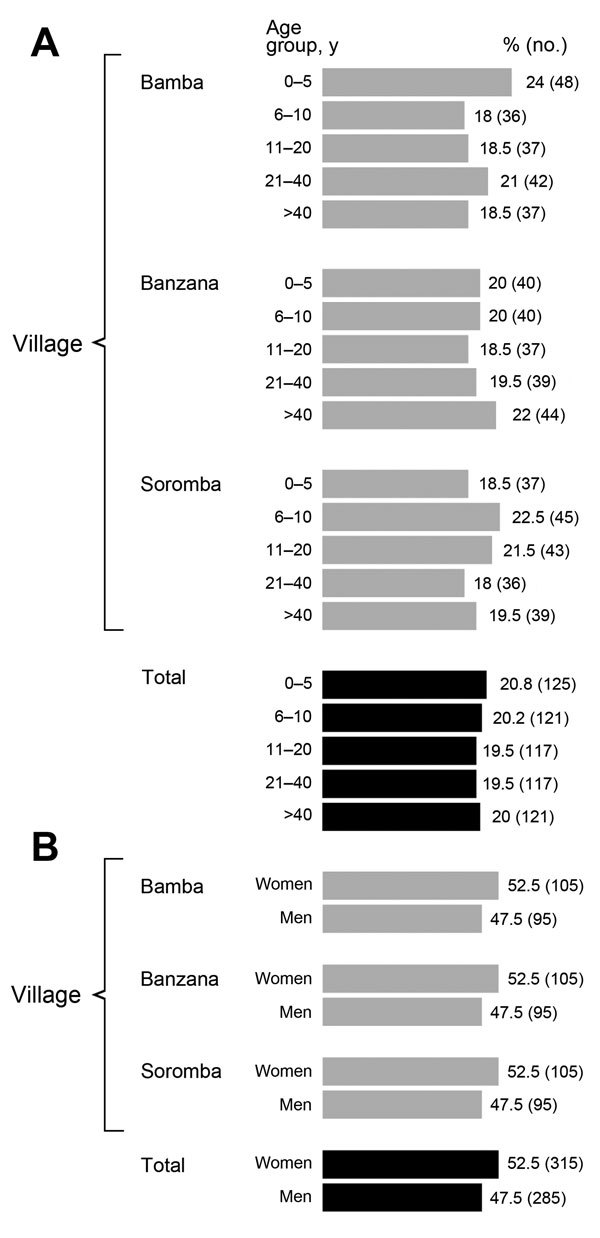
Demographic characteristics of study population in assessment of Lassa virus seroprevalence, southern Mali, 2015. A) Age; B) sex.

**A total of 600 participants were enrolled in this study, 200 per village, which represents 23.4%, 11.4%, and 4.1% of the populations of Soromba, Bamba, and Banzana, respectively. The average age of subjects enrolled was 21 years (range 7 months**–**83 years) (**Figure 2**). The sex ratio was slightly skewed toward female participants; 315 (52.5%) enrollees were female, versus 285 (47.5%) male (sex ratio 1.1) (**Figure 2**). The demographics of the study populations were essentially the same from each village and were representative of the population of Sibirila, which is primarily young (65% of inhabitants are <30 years old), with more female than male inhabitants. All study participants that were polled reported seeing rodents in their dwellings frequently and had an extensive history of febrile diseases without hemorrhagic manifestations.**

### Seroprevalence Rates

LASV IgM was detected in 4 samples, for an overall prevalence rate of 0.67%. All 4 IgM-positive samples were from female participants, ages 3, 11, 12, and 22 years (female-specific ratio of 4/315 [1.3%]). Three of the 4 samples reactive for IgM were also IgG positive. IgM-positive samples came from all 3 sites: 2 from Bamba, 1 from Banzana, and 1 from Soromba. The overall IgG seroprevalence for LASV across the 3 sites was 33.2% (199/600 samples) (Table 1). Overall village-specific LASV seroprevalence correlated with previous results of rodent surveys (*17*). The IgG seroprevalences for Bamba (44.0%; 95% CI 37.0%–51.2%) and Soromba (41.0%; 95% CI 34.1%–48.2%) were comparable and considerably higher than that for Banzana (14.5%; 95% CI 9.9%–20.2%) (Table 1).

**Table 1:** Lassa virus IgG seroprevalence by study site, southern Mali, 2015

Village	Male participants, no. positive/no. total (%)	Female participants, no. positive/no. total (%)	Combined seroprevalence, no. positive/no. total	Combined seroprevalence, % (95% CI)
Bamba	35/95 (37.2)	53/105 (50.5)	88/200	44.0 (37.0–51.2)
Banzana	14/95 (14.7)	15/105 (14.3)	29/200	14.5 (9.9–20.2)
Soromba	39/95 (41.1)	43/105 (41.0)	82/200	41 (34.1–48.2)
Total	88/285 (30.9)	111/315 (35.2)	199/600	33.2 (29.4–37.1)

Persons that were IgG positive were more likely to be older than those who were IgG negative (each 10+ years of age: RR 1.08; CI 1.02–1.14; p = 0.005) and were also more likely to be from Soromba than from Banzana (RR 2.8; CI 1.9–4.1; p<0.0001) but were not more likely to be from Bamba (p = 0.5). No differences in seropositivity were found by sex (p = 0.3) (Table 2). When analyzing IgG positivity as an outcome in a multivariate model, all significant variables from the univariate models (village [Banzana], age, and IgM OD) remained significant and at nearly the same magnitude of effect, suggesting that they are not confounding each other. Endpoint ELISA titers for most samples were relatively low, possibly because a nonhomologous recombinant LASV nucleocapsid antigen was used in the immunoassays (*20*). Three samples demonstrated titers of >6,400, and an additional 37 had titers of 1,600. The remaining samples had titers of 100 (n = 77) or 400 (n = 82).

**Table 2:** Lassa virus IgG seroprevalence by age group, in 3 villages, southern Mali, 2015

Age group, y	Village			Total no. positive/no. tested (%)
	Bamba, no. postive/no. tested (%)	Banzana, no. positive/no. tested (%)	Soromba, no. positive/no. tested (%)	
0–5	18/48 (37.5)	2/40 (*5*)	12/37 (32.4)	32/125 (25.6)
6–10	15/36 (41.7)	5/40 (12.5)	16/45 (35.6)	36/121 (29.8)
11–20	12/37 (32.4)	4/37 (10.8)	20/43 (46.5)	36/117 (30.8)
21–40	24/42 (57.1)	9/39 (23.1)	12/36 (33.3)	45/117 (38.5)
>40	19/37 (51.3)	9/44 (20.5)	22/39 (56.4)	50/120 (41.7)


## Discussion

LASV has most likely been present in southern Mali for several hundred years (*21*,*22*). However, until 2009, when an imported case of LF in a patient with travel history exclusive to Mali was diagnosed in the United Kingdom, LASV as well as LF were undocumented (*15*). Since then, despite increased awareness of the disease and ecologic studies defining the distribution of infected rodent reservoirs in the southern portions of the country, no additional case of LF has been observed or suspected. 

The apparent underrecognition of LASV infection in this region is likely multifactorial. The wide range of clinical features and nondescript symptoms that appear early in LASV infection impedes a diagnosis based strictly on clinical manifestations, even for experienced physicians (*23*–*25*). Studies in disease-endemic regions suggest that ≈20% of LASV infections lead to advanced and clinically severe disease manifestations. However, the classic indicators of LF may have easily been attributed to other infectious diseases, particularly malaria (as was the situation with the LF case imported to the United Kingdom), typhoid fever, or a variety of other etiologic agents known to have a high incidence in West Africa (*26*–*28*). In addition, the overall lack of confirmed cases in several West Africa countries where infected rodents and sporadic cases have been documented may be due to atypical or even attenuated clinical manifestations of these viruses, as has been suggested in recent nonhuman primate studies (*29*). Conceivably, the 80/20 ratio for LASV infection severity in many of the countries outside of the historical regions of endemicity could be even greater. Nevertheless, the absence of identifiable cases in this region is conspicuous, given that up to 50% of peridomestic rodents captured in some of these villages exhibit evidence of LASV infection (*17*).

To date, few LASV seroprevalence studies have been conducted outside of populations that reside in regions to which LASV is hyperendemic. With LASV IgG detectable in 33.2% of this study population, our results demonstrate that a high proportion of inhabitants in these 3 villages have been exposed to LASV and, by extension, indicate that a wider population in this region may also have been exposed. Village-specific prevalence rates were in accordance with the infection rates observed in rodents previously collected in this area. The IgG seroprevalences for Soromba and Bamba were 3-fold higher than that for Banzana, which is similar to infection rates observed in *Mastomys* rodents collected from these villages (*16*,*17*). 

IgG seropositivity was positively associated with age, which is not surprising given that the primary source for most infections would be infectious rodent reservoirs. The older a person is, the more likely they are to have had close contact with these animals. A substantial number of young children also demonstrated serologic evidence of previous exposure, which, because they were >7 months of age, are not likely to be false-positive results associated with maternal transfer of antibodies. Although seropositivity was higher in female participants, these results were not significant, suggesting that both sexes are equally infected in this region.

The prevalence reported here is, on average, slightly higher than (although similar to) results of previous human serosurveys conducted in disease-endemic and non–disease-endemic regions (*30*). In Liberia, Sierra Leone, Guinea, and Nigeria, LASV prevalence rates of 2%–52% by immunofluorescence assay and 10%–55% by ELISA have been documented (*20*,*23*,*31*–*35*). In countries where infected rodents, cases of LF, or both have only recently been documented, such as Benin, Ghana, and Côte d’Ivoire, seroprevalence rates among humans of 9.9%, 3.8%, and 20%, respectively, have been found by ELISA (*20*). 

Overall, the endpoint ELISA titers we report are low. However, these values are likely affected by the use of a nonregional LASV antigen from the lineage IV LASV strain Josiah. Although an ELISA based on a homologous LASV antigen may have been more appropriate, the kits we used (ReLASV), based on LASV Josiah from Sierra Leone, provided 2 strong advantages: 1) the standardized production and quality assessment ensures reliable and reproducible assays for current and future studies; and 2) the kits have been thoroughly evaluated in LASV-endemic areas for detection of LASV-reactive antibodies (a factors that strengthens the data presented here). A caveat regarding these kits is the potential for reduced endpoint titers. Emmerich et al. demonstrated reduced geometric mean titers in seropositive samples collected in Côte d’Ivoire (where LASV strain AV circulates) when diagnostic assays used antigen from strain Josiah rather than from strain AV (*20*). Full-genomic analysis demonstrates that LASV strain AV is genetically closest to the LASV strains from Mali, and, combined, may represent an emerging fifth lineage of LASV (*21*). 

Given these findings, the endpoint titers we found may be ≈2- to 4-fold lower than one might expect had the diagnostic antigen based on the LASV strains from Mali been used. Nonetheless, even if we considered the 77 samples with a serologic titer of 100 as equivocal, the overall seroprevalence in this study would still remain high (20.3%). Although the possibility exists that a certain proportion of persons with positive test results were exposed to a serologically cross-reactive arenavirus or were exposed during travel, the isolation of LASV from infected rodent reservoirs in many of these areas, combined with the observation that relatively few of these persons travel beyond their immediate geographic locations, argues against this.

In this study, we were unable to address possible risk factors associated with LASV seropositivity in Mali. A questionnaire was administered to study participants, but the data gained were limited. All participants reported seeing rodents frequently in their homes, with no apparent seasonality. No participants reported consuming rodents, although study staff observed adolescent boys cooking rodents over a fire on at least 1 trip to southern Mali (N. Sogoba, D. Safronetz, unpub. data). Social stigma may prevent persons from admitting to this practice.

 Furthermore, we were unable to provide insight into the clinical picture of LASV infection in this region. Not surprisingly, all participants in our study reported several episodes of febrile disease, although none with hemorrhagic manifestations or long-term sequelae, such as hearing loss, which is associated with LF. Because most of these clinically notable cases are likely attributed to malaria, the precise etiology of febrile diseases across Mali, in particular, in rural southern Mali, remains largely undefined. The Ebola virus outbreak in West Africa demonstrates why this diagnostic deficiency needs to be rapidly corrected.

In conclusion, the high seroprevalence rate we document highlights the need for increased surveillance for LASV in southern Mali. Further, prospective studies are required to define the clinical manifestations of LASV infection and LF in this region, as are follow-up studies to our work to determine incidence rates. Overall, these findings confirm that exposure to LASV is occurring in Mali, an area historically considered low risk for LF, and suggest that the annual incidence rate of LASV infection across West Africa may be higher than previously thought.

Technical Appendix Characteristics of villages in the rural Sibirilia commune of southern Mali and evidence of rodent infestation.
