# Supplementary material for: Lassa Virus Seroprevalence in Sibirilia Commune, Bougouni District, Southern Mali
Source: Emerg Infect Dis. 2016 Apr;22(4):657–63. doi: 10.3201/eid2204.151814 (PMC4806955; doi:10.3201/eid2204.151814)
Supplement: Technical Appendix — Characteristics of villages in the rural Sibirilia commune of southern Mali and evidence of rodent infestation. [file 15-1814-Techapp-s1.pdf]

DOI: http://dx.doi.org/10.3201/eid2204.151814

Lassa Virus Seroprevalence in Sibirilia Commune, Bougouni District, Southern Mali

Technical Appendix

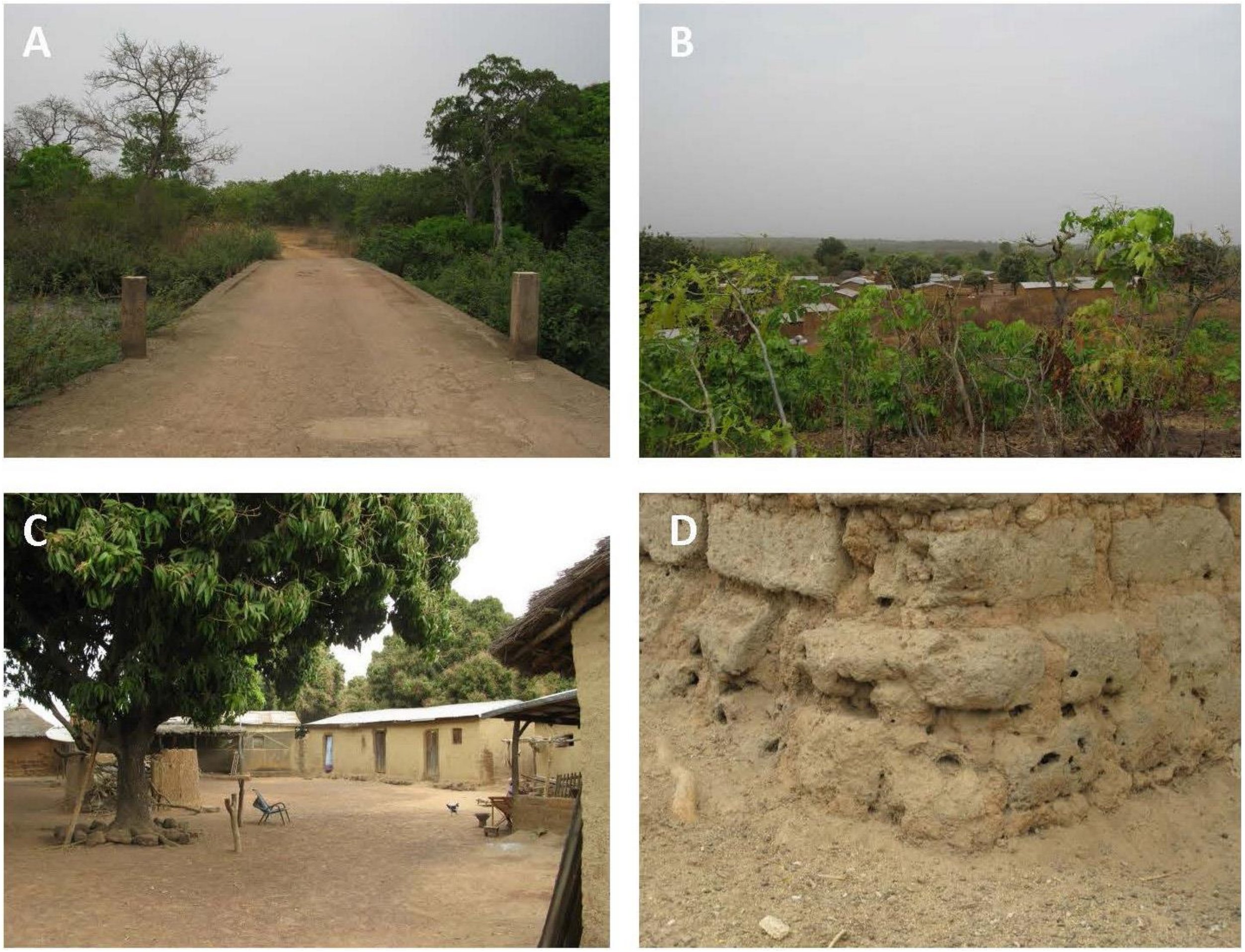

Technical Appendix Figure. Villages in the Sibirilia commune of southern Mali are predominantly rural and isolated by heavily wooded savannahs. A) The road to the village of Soromba; B) Soromba and surrounding area; C) typical housing in this region; D) evidence of rodent infestation. (Photos by David Safronetz with permission from village elders.)
